# Association of occupational heat exposure and colorectal cancer in the MCC-Spain study

**DOI:** 10.5271/sjweh.4082

**Published:** 2023-03-30

**Authors:** Alice Hinchliffe, Manolis Kogevinas, Antonio J Molina, Victor Moreno, Nuria Aragonés, Gemma Castaño-Vinyals, José Juan Jiménez Moleón, Inés Gómez Acebo, María Ederra, Pilar Amiano, Ana Molina-Barceló, Guillermo Fernandez-Tardon, Juan Alguacil, María-Dolores Chirlaque, Natalia Hernández-Segura, Beatriz Pérez-Gómez, Marina Pollan, Michelle C Turner

**Affiliations:** 1 Barcelona Institute for Global Health (ISGlobal), Barcelona, Spain; 2 Universitat Pompeu Fabra (UPF), Barcelona, Spain; 3 Consortium for Biomedical Research in Epidemiology & Public Health (CIBER Epidemiología y Salud Pública – CIBERESP), Madrid, Spain; 4 IMIM (Hospital del Mar Medical Research Institute), Barcelona, Spain; 5 The Research Group in Gene - Environment and Health Interactions (GIIGAS)/Institut of Biomedicine (IBIOMED), Universidad de León, León, Spain; 6 Unit of Biomarkers and Susceptibility, Oncology Data Analytics Program, Catalan Institute of Oncology (ICO), Hospital Duran i Reynals, L’Hospitalet de Llobregat, Barcelona, Spain; 7 Colorectal Cancer Group, ONCOBELL Program, Bellvitge Biomedical Research Institute (IDIBELL), L’Hospitalet de Llobregat, Barcelona, Spain; 8 Department of Clinical Sciences, Faculty of Medicine, University of Barcelona, Barcelona, Spain; 9 Epidemiology Section, Public Health Division, Department of Health of Madrid, Madrid, Spain; 10 Department of Preventive Medicine and Public Health, School of Medicine, University of Granada, Granada, Spain; 11 Instituto de Investigación Biosanitaria ibs.GRANADA, Granada, Spain; 12 Universidad de Cantabria – IDIVAL, Santander, Spain; 13 Instituto de Salud Pública y Laboral de Navarra, Navarra, Spain; 14 Instituto de Investigación Sanitaria de Navarra (IdisNa), Navarra, Spain; 15 Fundación Miguel Servet – Navarra biomed (FMS-NB), Navarra, Spain; 16 Ministry of Health of the Basque Government, Sub Directorate for Public Health and Addictions of Gipuzkoa, San Sebastian, Spain; 17 Biodonostia Health Research Institute, Epidemiology of Chronic and Communicable Diseases Group, San Sebastián, Spain; 18 Cancer and Public Health Unit, Foundation for the Promotion of Health and Biomedical Research (FISABIO-Public Health), Valencian Community, Spain; 19 Health Research Institute of Asturias (ISPA), Asturias, Spain; 20 University of Oviedo, Oviedo, Spain; 21 Centro de Investigación en Recursos Naturales, Salud y Medio Ambiente (RENSMA), Universidad de Huelva, Campus Universitario de El Carmen, Huelva, Spain; 22 Department of Epidemiology, Regional Health Council, IMIB-Arrixaca, Murcia University, Murcia, Spain; 23 Faculty of Health Sciences, Department of Biomedical Sciences, Area of Preventive Medicine and Public Health, Universidad de León, Spain; 24 Cancer and Environmental Epidemiology Unit, Department of Epidemiology and Chronic Diseases, National Center for Epidemiology, Carlos III Institute of Health, Madrid, Spain

**Keywords:** carcinogenesis, health effect, heat stress, occupational health, temperature, worker

## Abstract

**Objective:**

Heat exposure and heat stress/strain is a concern for many workers. There is increasing interest in potential chronic health effects of occupational heat exposure, including cancer risk. We examined potential associations of occupational heat exposure and colorectal cancer (CRC) risk in a large Spanish multi-case–­control study.

**Methods:**

We analyzed data on 1198 histologically confirmed CRC cases and 2690 frequency-matched controls. The Spanish job-exposure matrix, MatEmEsp, was used to assign heat exposure estimates to the lifetime occupations of participants. Three exposure indices were assessed: ever versus never exposed, cumulative exposure and duration (years). We estimated odds ratios (OR) and 95% confidence intervals (CI) using unconditional logistic regression adjusting for potential confounders.

**Results:**

Overall, there was no association of ever, compared with never, occupational heat exposure and CRC (OR 1.09, 95% CI 0.92–1.29). There were also no associations observed according to categories of cumulative exposure or duration, and there was no evidence for a trend. There was no clear association of ever occupational heat exposure and CRC in analysis conducted among either men or women when analyzed separately. Positive associations were observed among women in the highest categories of cumulative exposure (OR 1.81, 95% CI 1.09–3.03) and duration (OR 2.89, 95% CI 1.50–5.59) as well as some evidence for a trend (P<0.05).

**Conclusion:**

Overall, this study provides no clear evidence for an association between occupational heat exposure and CRC.

Colorectal cancer (CRC) is the third most common cancer globally, accounting for approximately 10.0% of all new cancer cases, and the second most fatal cancer, responsible for 9.4% of cancer deaths worldwide (1). In Spain, CRC was the most commonly diagnosed cancer in 2020, with an estimated 40 441 new cases, or 14.3% of all newly diagnosed cancers, and there were 16 470 deaths (1). Established risk factors for CRC include older age, ethnicity, family history, obesity, lack of physical activity, tobacco smoking, alcohol intake, and consumption of processed meat (2). There are also some occupational exposures or agents that are suspected as possible colorectal carcinogens, including asbestos, night shift work, and occupational exposure as a firefighter (firefighters are exposed to a range of hazardous agents), although the strength of the evidence remains limited (3–5).

In many occupations, heat exposures are common (6). It is estimated worldwide that more than 1 billion workers have been exposed to high heat episodes, and this number is expected to rise (7). Acute heat stress related conditions, including heat stroke and death, are well-recognized. When temperatures rise, the body’s thermoregulatory system can become overwhelmed, causing the core temperature to increase (8). This can be further exacerbated among workers wearing heavy clothing and engaging in high levels of physical activity, also increasing metabolic heat production (8). Heat stress can have adverse effects on a range of body tissues and organs, including the brain, cardiorespiratory system, intestines, and digestive tract which can persist for years following injury (7). For example, high incidence rates of chronic kidney disease (CKD) have been described among workers exposed to high temperatures (9). This is thought to be due at least in part to repeated episodes of dehydration which can cause acute kidney injury, that manifests chronically (9).

Heat stress has been shown to exhibit some key characteristics of human carcinogens (10–12). Workers exposed to heat have also been observed to have higher dietary salt intake to compensate for excess salt excreted through increased sweating (13). Ingestion of salt and salted/salty foods has been linked to an increased risk of certain gastrointestinal cancers, including stomach and colorectal cancer (14, 15). Heat-exposed workers may also be exposed to various other occupational agents and the biological response to heat exposure, including increased skin permeability and higher respiration rate, can potentially exacerbate chemical absorption and toxicity to other agents. Gender differences in thermoregulation have also been noted, with women performing work at the same level as men experiencing greater core temperature rises due to lower body mass, higher fat content and lower sweat output (16).

Some previous studies have investigated associations between occupational titles and risk of different cancers. Among workers in some heat-exposed occupations, positive associations have been reported for breast (17, 18), prostate (19), and gastrointestinal cancers (18, 20, 21). However, studies have also reported no associations with cancer risk (18, 22).

There are also studies evaluating occupational heat exposure more specifically and different gastrointestinal cancers. A cohort study of Finnish women (23) (N=413 877) undertaken between 1971 and 1995, converted occupations taken from the 1970 census of Finland to job codes according to the Nordic Classification of Occupations and the International Classification of Occupations and then assigned heat exposure estimates using the Finnish job exposure matrix (FINJEM). The study reported no associations between occupational heat exposure and various gastrointestinal cancers, including colon and rectal cancer. The PANESOES study, undertaken in Spain between 1995 and 1999, is a hospital-based case–control study designed to explore the influence of major lifestyles and diet on the risk of stomach, pancreatic and esophageal cancer. Information on sociodemographic, lifestyle and occupational factors (main occupation, job title, number of years worked) was collected in face-to-face interviews, and occupational heat exposure estimates were subsequently assigned using the FINJEM. No significant associations were observed between occupational heat exposure and the risk of stomach (24) (399 cases, 455 controls), pancreatic (25) (161 cases, 455 controls) or esophageal cancer (26) (185 cases, 285 controls). An earlier Spanish case–control study (27), undertaken between 1992 and 1995 also observed no association between occupational heat exposure and pancreatic cancer risk (185 cases, 264 controls). In contrast, a case–control study conducted in Finland (28) (595 cases, 1622 controls) between 1984 and 1987 found positive associations with pancreatic cancer. The study collected lifelong work histories from the next-of-kin using postal questionnaires. Occupational heat exposure estimates were then assigned by an experienced industrial hygienist with the use of a job exposure matrix (JEM) created in the United Kingdom. Other studies on occupational heat exposure and several other cancers have also had mixed results (29–33).

Limitations of previous studies include insufficient statistical power due to small numbers of cancer cases, cross-sectional job assessment, or assessment only considering the longest worked occupation. In other recent work, we reported some evidence for positive associations of lifetime occupational heat exposure and female breast cancer, but not prostate cancer (34, 35). Further studies are needed to better examine potential associations between occupational heat exposure and cancer risk, including studies of digestive organs.

Here we analyzed the potential association of lifetime occupational heat exposure and CRC risk in a large-scale population-based multi-case–control study, expanding on the limited current knowledge and building on other previous work.

## Methods

### Study data

The MCC-Spain study (36) (www.mccspain.org) is a population-based multi-case–control study carried out between 2008 and 2013 including cases of colorectal, breast, prostate and stomach cancers and chronic lymphocytic leukemia and population controls from the catchment area of 23 hospitals in 12 Spanish administrative regions. The study included 2140 newly diagnosed CRC cases and 3950 population controls. Inclusion criteria were age 20–85 years, residence in the catchment area for ≥6 months prior to recruitment, having no prior history of CRC and ability to answer the epidemiological questionnaire. Controls, frequency-matched to cases by age (in 5-year age groups), sex and region, were randomly selected from the administrative records of selected primary care health centers located within the hospitals’ catchment areas and were invited to participate through the telephone. Response rates varied by center and on average were 68% among cases and 54% among controls. Detailed occupational information for all jobs held for ≥1 year, along with a thorough personal and family medical history and information on lifestyle factors was obtained through face-to-face interviews performed by trained personnel.

The MCC-Spain study followed the national and international directives on ethics and data protection [declaration of Helsinki and Spanish law on confidentiality of data (Ley Organica 15/1999 de 13 Diciembre de Proteccion de Datos de carácter personal LOPD)]. All subjects who agreed to participate and met the eligibility criteria gave written informed consent before participating in the study. The ethics committees of all participating institutions approved the protocol of MCC-Spain.

### Occupational heat exposure assessment

Two industrial hygienists blinded to the case–control status of participants coded job titles according to the Spanish National Classification of Occupations (CNO-94). Estimates of the proportion of workers exposed to heat (P) and the level of exposure (L) were subsequently assigned to each job using a Spanish JEM (MatEmEsp) (37), which covered the period 1996–2005. For heat, the level of exposure (L) is considered as the average yearly proportion of working time with heat stress. In MatEmEsp, occupational heat exposure is defined as continual exposure to natural or artificial heat above specific wet bulb globe temperature (WBGT) indices determined in ISO 7243, an international standard for the assessment of thermal environments (38). The WBGT thresholds for ‘safe’ hourly continuous work range from 31°C for light intensity work to 25.5°C for very heavy intensity work. MatEmEsp provides heat exposure estimates for occupations in which ≥5% of workers are exposed to temperatures exceeding the WBGT indices applicable to each situation, as verified with quantitative data or from the estimation of industrial hygienists. Estimates of heat exposure in MatEmEsp are based on those in FINJEM, and were extensively adapted to Spanish working conditions by local experts. A panel of five actively employed industrial hygienists with extensive experience in company-based industrial hygiene measurements in Spain revised exposure estimates from FINJEM to more accurately represent those for each job title amongst Spanish workers.

### Statistical analysis

For the present study, data from a total of 1198 CRC cases and 2690 controls was analyzed. We excluded a subset of 176 controls and 271 cases here as their occupational history was collected using a somewhat different protocol as part of pilot testing. We additionally excluded participants who were exclusively housewives, as housework was not included in the JEM (244 controls, 138 cases), participants with a previous personal history of cancer (283 controls, 157 cases), and participants who had missing occupational information, including missing occupational codes or start/finish years (148 controls, 127 cases).

Distributions of potential risk factors among CRC cases and controls, and controls ever and never occupationally exposed to heat, were compared using Wilcoxon rank sum and chi-squared tests. Unconditional logistic regression models were used to estimate odds ratios (OR) and 95% confidence intervals (95% CI) for the association between CRC risk and three lifetime occupational heat exposure indices: ever versus never, cumulative exposure, and duration of exposure. Colon and rectal cancer cases were also analyzed separately.

Ever occupational heat exposure was defined *a priori* as having held ≥1 job with a P≥25% and with an exposure duration of ≥1 year. We deemed participants with a P of 5–25% or with occupational heat exposure for <1 year to have uncertain exposure and, to balance sensitivity and specificity, we excluded them from the analysis (355 controls, 233 cases), as similar to previous work (34, 35). To allow for a possible cancer latency period, an *a priori* lag of five years was applied to all analyses. All exposures occurring in the five years before diagnosis date for cases and interview date for controls were therefore not included in the main analysis. Participants only exposed in the five years before diagnosis/interview date were considered unexposed.

Lifetime cumulative exposure was calculated as the sum of the product of P, L, and duration of occupational heat exposure for all jobs with a P≥25% according to the above definition and was categorized into tertiles according to the distribution among exposed controls.

Duration of occupational heat exposure was defined as the sum of the duration of occupational heat exposure for all jobs with a P≥25% according to the above definition. Overlapping jobs held during the same time period were considered part-time, and duration of these jobs was split equally between them. Duration was categorized into >0– <15 years, ≥15–<30 years and ≥30 years, based on approximate tertiles according to the distribution among exposed controls. The reference group for all analyses was the group of workers that were never exposed to occupational heat.

In minimally-adjusted models, there were variables included for age (as a continuous variable), sex, region (11 Spanish regions from which CRC cases and matched controls participants were recruited), and education [less than primary, primary (6–16 years old), secondary (16–18 years old), university]. A directed acyclic graph and *a priori* knowledge were used to identify other potential confounders. In fully-adjusted analysis, models were also adjusted for cigarette smoking (never, ex-, and current smoker), family history of CRC in a first degree relative (yes/no/missing), body mass index [BMI (kg/m^2^)] within one year before diagnosis/interview date, and self-reported physical activity at work (sedentary, low active, moderately active, vigorously active, extremely active). We created a missing indicator as a third category for family history of CRC to include participants with missing information. We excluded participants with missing information on any of the other variables (54 controls, 16 cases). Ordinal variables were taken as continuous to test for linear trends, using unexposed participants as the reference category.

We additionally assessed the impact of further adjusting models for other potential confounders, including leisure-time physical activity (inactive, a little active, moderately active, and very active) (both instead of physical activity at work, and in addition to physical activity at work) [while greater levels of physical activity at work can be detrimental, raising the risk of heat stress due to increased metabolic heat production, higher levels of physical activity and physical fitness in general may be protective of heat stress as well as of CRC (39)], diet and alcohol consumption (constructed of scores assigned according to adherence to the World Cancer Research Fund recommendations for cancer prevention) (40) available for most included participants (2401 controls, 1060 cases), and night shift work (ever versus never working a schedule that involved working partly or entirely between 00:00 and 06:00 hours, ≥3 times per month) (41). We also evaluated the effect of adjusting models for socioeconomic score (constructed using participants’ education level, social class by occupation and parents’ socioeconomic status) as an alternative to education. Further analyses were also conducted according to strata of sex, cigarette smoking and education. We investigated the impact of timing of last heat exposure being ≥5–<10, ≥10–<20 and ≥20 years before the diagnosis/interview date.

In a further attempt to comprehensively account for other occupational exposures in analysis here, we also assessed the potential confounding and effect modifying effects of other occupational agents. For this analysis, ever occupational exposure was defined as having ever held ≥1 job with a P≥5% for a duration of ≥1 year, as exposure prevalence for other occupational agents was low. We assessed occupational agents which were contained in MatEmEsp, and for which there were sufficient participants exposed to heat and the other agents including any metal (lead, cadmium, chromium, nickel, iron), solvent (aliphatic and alicyclic hydrocarbons, aromatic hydrocarbons, chlorinated hydrocarbons, and other organic solvents), pesticide (2,4-D, atrazine, captan, chlorpyrifos, dicuat, diuron, endosulfan, methomyl, pyrethrin, tiram), polycyclic aromatic hydrocarbons (PAHs), and detergents. Some studies have previously linked some agents with CRC risk (42–45), although the evidence is uncertain.

Finally, as part of the sensitivity analyses, we explored the effect of *a priori* decisions on the results. In addition to the default P≥25%, exposure duration of ≥1 year and lag period of five years, we analyzed alternative threshold combinations. We investigated P thresholds of ≥5% and ≥50%, an exposure duration of ≥5 years and lag periods of one and ten years.

All analyses were conducted using Stata 17, StataCorp, College Station, TX, USA.

## Results

Table 1 shows distributions of characteristics of the 1198 included cases and 2690 controls. Cases were somewhat older than controls [65.6, standard deviation (SD) 11.2, versus 61.5, SD 11.8, years], were more often male, had a lower level of educational attainment, were less likely to be current smokers, more likely to have a family history of CRC in a first degree relative, and had a higher level of physical activity at work.

**Table 1:** Distributions of risk factors among colorectal cancer cases and controls. [SD=standard deviation]. Wilcoxon rank-sum for continuous and chi-square for categorical. Numbers may differ due to missing values.

	Cases (N=1198)		Controls (N=2690)		P-values
	
	N (%)	Mean (SD	N (%)	Mean (SD)	
Age		65.6 (11.2)		61.5 (11.8)	<0.001
Sex					
Males	776 (64.8)		1324 (49.2)		
Females	422 (35.2)		1366 (50.8)		<0.001
Region					
Madrid	152 (12.7)		568 (21.1)		
Barcelona	275 (23.0)		565 (21.0)		
Navarra	87 (7.3)		201 (7.5)		
Guipuzcoa	79 (6.6)		275 (10.2)		
Leon	234 (19.5)		277 (10.3)		
Asturias	52 (4.3)		145 (5.4)		
Murcia	19 (1.6)		29 (1.1)		
Huelva	40 (3.3)		115 (4.3)		
Cantabria	91 (7.6)		271 (10.1)		
Valencia	60 (5.0)		106 (3.9)		
Granada	109 (9.1)		138 (5.1)		<0.001
Education					
Less than primary school	319 (26.6)		410 (15.2)		
Primary school	427 (35.6)		773 (28.7)		
Secondary school	281 (23.5)		837 (31.1)		
University	171 (14.3)		670 (24.9)		<0.001
Smoking					
Never smoker	469 (39.2)		1133 (42.1)		
Ex-smoker	560 (46.7)		982 (36.5)		
Current smoker	169 (14.1)		575 (21.4)		<0.001
Family history of colorectal cancer					
No	943 (78.7)		2341 (87.0)		
Yes	204 (17.0)		233 (8.7)		
Missing	51 (4.3)		116 (4.3)		<0.001
Body mass index (kg/cm^2^)		27.4 (4.5)		26.5 (4.5)	<0.001
Physical activity at work					
Sedentary	126 (10.5)		521 (19.4)		
Low active	134 (11.2)		391 (14.5)		
Moderately active	388 (32.4)		855 (31.8)		
Vigorously active	341 (28.5)		586 (21.8)		
Extremely active	209 (17.5)		337 (12.5)		<0.001

Characteristics of controls ever (N=984) and never (N=1706) having occupational heat exposure are presented in the supplementary material (www.sjweh.fi/article/4082), table S1. Controls ever having occupational heat exposure were somewhat older (63.8, SD 11.1, versus 60.2, SD 11.9, years), male, more likely to have ever smoked cigarettes, less well educated, and had a higher level of physical activity at work.

Overall, 51% of cases and 37% of controls were classified as being ever occupationally exposed to heat (table 2). Occupations with the highest heat exposure [level (%)] included operators of stationary industrial installations, blacksmiths and smiths, and boiler and steam engine operators (supplementary table S2). The most common heat-exposed jobs included waiters, waitresses and bartenders, agricultural workers, cooks, bricklayers, and laborers in manufacturing industries. Amongst those exposed, the average duration of exposure was 23 (SD 16.6) years and the average lifetime cumulative exposure was 587 (P×L×duration, SD 651, years).

**Table 2:** Associations between occupational heat exposure and colorectal cancer [OR=odds ratio; CI=confidence interval]

	Colorectal cancer			Colon cancer ^a^		Rectal cancer ^a^	
		
	Cases/Controls (N)	OR (95% CI) ^b^	OR (95% CI) ^c^	Cases/Controls (N)	OR (95% CI) ^c^	Cases/Controls (N)	OR (95% CI) ^c^
Never heat exposure	585/1706	1 (ref)	1 (ref)	362/1706	1 (ref)	220/1706	1 (ref)
Ever heat exposure	613/984	1.18 (1.00–1.38)	1.09 (0.92–1.29)	341/984	1.00 (0.81–1.22)	263/984	1.23 (0.97–1.56)
Lifetime cumulative exposure ^d^							
Low	153/328	1.21 (0.97–1.52)	1.15 (0.92–1.45)	82/328	1.02 (0.77–1.35)	68/328	1.37 (1.00–1.88)
Medium	198/328	1.14 (0.91–1.42)	1.06 (0.85–1.34)	117/328	1.04 (0.79–1.36)	78/328	1.07 (0.77–1.48)
High	262/328	1.17 (0.94–1.47)	1.03 (0.82–1.30)	142/328	0.93 (0.71–1.24)	117/328	1.23 (0.90–1.70)
P-trend		0.12	0.70		0.76		0.23
Duration (years) ^e^							
>0–<15	223/451	1.19 (0.97–1.45)	1.13 (0.92–1.38)	130/451	1.06 (0.83–1.35)	88/451	1.21 (0.91–1.62)
≥15–<30	119/185	1.24 (0.95–1.62)	1.11 (0.84–1.47)	61/185	0.96 (0.68–1.35)	58/185	1.39 (0.97–2.01)
≥30	271/348	1.13 (0.90–1.41)	1.00 (0.80–1.27)	150/348	0.93 (0.70–1.23)	117/348	1.15 (0.83–1.58)
P-trend		0.18	0.82		0.60		0.26

a The numbers of colon and rectal cancers may not equal the total number of colorectal cancers as tumour site was unknown in some cases.

b Minimally adjusted models adjusted for age, sex, region, and education.

c Fully adjusted models adjusted for age, sex, region, education, cigarette smoking, family history of colorectal cancer, BMI, and occupational physical activity.

d P×L×duration in years, cut points for all analyses: low (>0–<157), medium (≥157–<588), and high (≥588).

e Based on approximate tertiles according to the distribution amongst exposed controls.

In minimally-adjusted models, there was a weak positive association of ever, compared with never, occupational heat exposure and CRC (OR 1.18, 95% CI 1.00–1.38), and in categories of lifetime cumulative exposure and duration, although no trends were observed (table 2). In fully-adjusted models, there was no evidence for an association of ever occupational heat exposure and CRC (OR 1.09; 95% CI 0.92–1.29). No discernible trends were observed across categories of lifetime cumulative exposure and duration, and there was no evidence for an exposure–response trend. In an analysis of colon and rectal cancer cases separately, there were some weakly elevated OR observed for ever occupational heat exposure and rectal (OR 1.23; 95% CI 0.97–1.56) but not colon cancer, as well as in some categories of lifetime cumulative exposure and duration, although there were no discernible trends.

In sensitivity analysis further adjusting models for leisure-time physical activity, diet, alcohol consumption, and night shift work, findings were generally unchanged (not shown). Findings were also unchanged when adjusting models for socioeconomic score as an alternative to education.

Table 3 shows the associations between occupational heat exposure and CRC risk stratified by sex. There was no clear association of ever occupational heat exposure and CRC risk among either men or women. There were some positive associations observed among women in the highest categories of cumulative exposure (OR 1.81, 95% CI 1.09–3.03) and duration (OR 2.89, 95% CI 1.50–5.59) and significant trends (P<0.05). There were also significant interactions by gender (P<0.05). However, findings were based on small numbers of exposed women (62% of male CRC cases were ever exposed to occupational heat compared with 32% of female CRC cases) and maybe due to chance. In analyses stratified by either cigarette smoking or education, there were no clear associations observed and no evidence for an interaction (tables 4 and 5).

**Table 3:** Associations between occupational heat exposure and colorectal cancer stratified by sex [OR=odds ratio; CI=confidence interval]. All models are adjusted for age, region, education, cigarette smoking, family history of colorectal cancer, body mass index, and occupational physical activity.

	Males		Females		P-values for interaction
	
	Cases/Controls (N)	OR (95% CI)	Cases/Controls (N)	OR (95% CI)	
Never heat exposure	295/643	1 (ref)	290/1063	1 (ref)
Ever heat exposure	481/681	1.02 (0.82–1.27)	132/303	1.28 (0.97–1.70)	0.37
Lifetime cumulative exposure ^a^					
Low	106/166	1.22 (0.91–1.65)	47/162	1.01 (0.69–1.48)	
Medium	149/230	0.91 (0.68–1.22)	49/98	1.42 (0.94–2.14)	
High	226/285	0.93 (0.70–1.24)	36/43	1.81 (1.09–3.03)	
P-trend		0.54		0.01	0.03
Duration (years) ^b^					
>0–<15	143/219	1.14 (0.87–1.51)	80/232	1.12 (0.81–1.54)	
≥15–<30	94/134	1.01 (0.72–1.41)	25/51	1.29 (0.74–2.23)	
≥30	244/328	0.91 (0.69–1.19)	27/20	2.89 (1.50–5.58)	
P-trend		0.43		0.005	0.02

a P×L×duration in years, cut points based on those of the overall population.

b Based on approximate tertiles according to the distribution amongst exposed controls.

**Table 4:** Associations between occupational heat exposure and colorectal cancer stratified by cigarette smoking. [OR=odds ratio; CI=confidence interval]. All models are adjusted for age, sex, region, education, family history of colorectal cancer, BMI and occupational physical activity.

	Never smokers		Ever smokers		P-values for interaction
	
	Cases/Controls (N)	OR (95% CI)	Cases/Controls (N)	OR (95% CI)	
Never heat exposure	246/767	1 (ref)	339/939	1 (ref)	
Ever heat exposure	223/366	1.26 (0.97–1.64)	390/618	1.01 (0.81–1.27)	0.47
Lifetime cumulative exposure ^a^					
Low	57/123	1.24 (0.86–1.80)	96/205	1.07 (0.80–1.43)	
Medium	77/119	1.37 (0.96–1.96)	121/209	0.94 (0.70–1.27)	
High	89/124	1.17 (0.80–1.70)	173/204	1.02 (0.75–1.38)	
P-trend		0.18		0.99	0.67
Duration (Years) ^b^					
>0–<15	95/180	1.28 (0.94–1.75)	128/271	1.03 (0.79–1.35)	
≥15–<30	46/62	1.49 (0.95–2.33)	73/123	0.91 (0.64–1.30)	
≥30	82/124	1.08 (0.73–1.60)	189/224	1.05 (0.78–1.41)	
P-trend		0.35		0.87	0.69

a P×L×duration in years, cut points based on those of the overall population.

b Based on approximate tertiles according to the distribution amongst exposed controls.

**Table 5:** Associations between occupational heat exposure and colorectal cancer stratified by education. [OR=odds ratio; CI=confidence interval]. All models are adjusted for age, sex, region, education, family history of colorectal cancer, BMI and occupational physical activity.

	Primary school or less		Secondary school or more		P-values for interaction
	
	Cases/Controls (N)	OR (95% CI)	Cases/Controls (N)	OR (95% CI)	
Never heat exposure	273/532	1 (ref)	312/1174	1 (ref)	
Ever heat exposure	473/651	1.11 (0.89–1.38)	140/333	1.16 (0.89–1.50)	0.58
Lifetime cumulative exposure ^a^					
Low	80/154	1.02 (0.74–1.42)	73/174	1.29 (0.94–1.78)	
Medium	155/220	1.12 (0.84–1.48)	43/108	1.10 (0.74–1.66)	
High	238/277	1.16 (0.88–1.53)	24/51	0.89 (0.52–1.52)	
P-trend		0.26		0.75	0.40
Duration (Years) ^b^					
>0–<15	143/248	1.07 (0.82–1.40)	80/203	1.23 (0.90–1.67)	
≥15–<30	87/118	1.17 (0.83–1.65)	32/67	1.20 (0.74–1.93)	
≥30	243/285	1.12 (0.85–1.48)	28/63	0.94 (0.57–1.55)	
P-trend		0.39		0.65	0.31

a P×L×duration in years, cut points based on those of the overall population

b Based on approximate tertiles according to the distribution amongst exposed controls

Findings were generally unchanged when adjusting models for ever/never exposure to other occupational agents: metals, solvents, pesticides, PAH, and detergents (not shown). The prevalence of participants ever occupationally exposed to both heat and other occupational agents ranged from 9% for solvents up to 32% for metals. There was also no evidence for effect modification of associations of occupational heat exposure and CRC according to exposure to other occupational agents (supplementary tables S3–7).

Findings were generally unchanged when stratified by time since last heat exposure (supplementary table S8). When using different P-thresholds, exposure durations and lag periods, as part of sensitivity analyses, results were also similar (supplementary tables S9–11).

## Discussion

In this large-scale population-based multi-case–control study, we found no evidence overall for an association between occupational heat exposure and CRC risk and found no discernible trend across categories of lifetime cumulative exposure or duration. There were positive associations among women in the highest categories of lifetime cumulative exposure and duration, as well as significant trends, although findings were based on small numbers of exposed women.

In previous work on occupational heat exposure and female breast (34) and prostate cancer (35), positive associations were observed for female breast cancer, but not for prostate cancer. Women ever occupationally exposed to heat had an increased risk of breast cancer (OR 1.22, 95% CI 1.01–1.46) in an analysis of 1389 breast cancer cases and 1434 controls in the MCC-Spain study. There were also significant trends according to categories of lifetime cumulative exposure and duration. In contrast, there was no evidence for an association between occupational heat exposure and prostate cancer in a pooled analysis of data from three international case–control studies, including MCC-Spain (36, 46, 47).

The somewhat stronger associations of occupational heat exposure and CRC among females likely reflects a chance finding due to the small numbers of exposed women in the analysis. There are also differences in occupational heat exposure profiles and in the most common heat-exposed jobs among men and women. The average lifetime cumulative exposure and duration for males was approximately twice that of females. The most common heat-exposed jobs among males included bricklayers, carpenters, agricultural workers, and construction workers, while among females they included cooks, laborers in manufacturing industries, helpers and cleaners and launderers and ironers, with males more commonly exposed to outdoor heat, while female heat exposure was mostly indoors. There could also be differences in other occupational co-exposures between male and females as well as differences in physical traits and physiology. Studies have shown males have a shorter heat acclimatization period than females (48, 49). The thermoregulatory responses of women can also vary over the menstrual cycle and at menopause due to the influences of reproductive hormones (50). It is also possible that residual confounding contributed to the differing results between men and women. Although we were able to control for multiple confounders here, it is possible that some confounding effect remained, due to imperfect measurement of the confounding variables or inaccurate adjustment. Unmeasured potential confounding factors may also impact findings. In an attempt to explore potential residual confounding, analysis among females was also performed with further adjustment for other lifestyle factors variables (above), though findings were generally unchanged (not shown).

This study has several strengths. We were able to examine associations using a large number of histologically confirmed CRC cases and controls frequency matched by age, sex and region. Participants were from multiple regions of Spain and provided detailed lifetime occupational histories, including a wide range of occupations, making results more generalizable. The availability of lifetime occupational history allowed us to examine the exposure of participants over the entire working life. Using a JEM allowed us to apply standardized heat exposures to all participants, limiting the chance of recall bias. The collection of information on potential confounding factors allowed us to adjust our results comprehensively. This study contributes to the current limited evidence on occupational heat exposure and cancer risk, in particular CRC.

The study also has some limitations. The development of various exposure indices and definition of ever occupational heat exposure could have caused some non-differential misclassification bias, although the effect was likely minimal as results were generally unchanged in sensitivity analyses with a range of categories. Additionally, multiple comparisons were made without adjustment, and some of the results could have occurred by chance (above). Heat exposure estimates were assigned to job titles rather than to individual participants, and exposure variability between workers in the same job is not considered. This generally leads to reduced precision but less bias (51). In MatEmEsp, heat estimates only cover 1996–2005, and there maybe misclassification particularly for more historical jobs where exposure information is lacking. Some job titles were unspecific, which could have caused further misclassification errors. The use of a JEM also allowed us to explore additional chemical and physical exposures. However, the prevalence of ever exposure to other occupational exposures was low, including among heat-exposed workers and there was no evidence for potential confounding or effect modification observed here. In previous work on prostate cancer, there was some evidence for stronger positive associations with occupational heat exposure observed among participants ever occupationally exposed to PAH, and there was some evidence of an interaction, though the prevalence of exposure in PAH expoure was generally low or uncertain (P range 5–25%) (35).

As global average temperatures rise and the frequency and severity of extreme weather events, such as heat waves, increases due to climate change, occupational heat exposures are projected to intensify (52, 53). This will likely affect both outdoor and indoor occupations with inadequate temperature control. There is a need for further studies of cancer to build on the limited evidence currently available. Subsequent studies may consider examination of the most highly exposed workers, and improving exposure estimates, including both JEM-based and personal measurements of workers, as well as of potential biological pathways and mechanisms that could link heat exposure to cancer. Some slightly higher OR were observed when analyzing rectal cancer separately, possibly due to morphological, physiological, and biochemical differences between the colon and rectum (54), further research according to subsite maybe warranted. Further studies to consider potential sex differences as well as of interactions with other occupational co-exposures would also be of interest.

### Concluding remarks

This study provides little evidence overall for an association between occupational heat exposure and CRC risk. Further research to investigate chronic health effects of occupational heat exposure are warranted.

### Conflicts of interest

The authors declare no conflicts of interest.

## Supplementary material

Supplementary material
