# Supplementary material for: Association of occupational heat exposure and colorectal cancer in the MCC-Spain study
Source: Scand J Work Environ Health. 2023 Mar 30;49(3):211–21. doi: 10.5271/sjweh.4082 (PMC10621896; doi:10.5271/sjweh.4082)
Supplement: Supplementary material [file SJWEH-49-211-S001.pdf]

Association of occupational heat exposure and colorectal cancer in the MCC-Spain study[1]

by Alice Hinchliffe, MSc, Manolis Kogevinas, PhD, Antonio J Molina, PhD, Victor Moreno, PhD, Nuria Aragonés, PhD, Gemma Castaño-Vinyals, PhD, José Juan Jiménez Moleón, PhD, Inés Gómez Acebo, PhD, María Ederra, PhD, Pilar Amiano, PhD, Ana Molina-Barceló, PhD, Guillermo Fernandez-Tardon, PhD, Juan Alguacil, PhD, María-Dolores Chirlaque, PhD, Natalia Hernández-Segura, MSc, Beatriz Pérez-Gómez, PhD, Marina Pollan, PhD, Michelle C Turner, PhD [2]

1. Supplementary material.
2. Correspondence to: Michelle C. Turner, ISGlobal, Barcelona Institute for Global Health, Parc de Recerca Biomèdica de Barcelona, Doctor Aiguader, 88 Barcelona 08003 Spain. [E-mail: michelle.turner@isglobal.org] ORCID: 0000-0002-6431-1997

Table S1. Distributions of risk factors among controls ever and never occupationally exposed to heat

	Ever heat (N=984)	Never heat (N=1,706)	*p-values*
	N(%)	N(%)	
**Age; mean (SD)**	63.8 (11.1)	60.2 (11.9)	<0.001
**Sex**			
Males	681 (69.2)	643 (37.7)	
Females	303 (30.8)	1,063 (62.3)	<0.001
**Region**			
Madrid	139 (14.1)	429 (25.2)	
Barcelona	262 (26.6)	303 (17.8)	
Navarra	57 (5.8)	144 (8.4)	
Guipuzcoa	69 (7.0)	206 (12.1)	
Leon	126 (12.8)	151 (8.9)	
Asturias	57 (5.8)	88 (5.2)	
Murcia	15 (1.5)	14 (0.8)	
Huelva	56 (5.7)	59 (3.5)	
Cantabria	111 (11.3)	160 (9.4)	
Valencia	29 (3.0)	77 (4.5)	
Granada	63 (6.4)	75 (4.4)	<0.001
**Education**			
Less than primary school	251 (25.5)	159 (9.3)	
Primary school	400 (40.7)	373 (21.9)	
Secondary school	221 (22.5)	616 (36.1)	
University	112 (11.4)	558 (32.7)	<0.001
**Smoking**			
Never smoker	366 (37.2)	767 (45.0)	
Ex-smoker	402 (40.9)	580 (34.0)	
Current smoker	216 (22.0)	359 (21.0)	<0.001
**Family history of colorectal cancer**			
No	864 (87.8)	1,477 (86.6)	
Yes	76 (7.7)	157 (9.2)	
Missing	44 (4.5)	72 (4.2)	0.41
**BMI (kg/cm2); mean (SD)**	27.4 (4.4)	25.9 (4.4)	<0.001
**Physical activity at work**			
Sedentary	78 (7.9)	443 (26.0)	
Low active	99 (10.1)	292 (17.1)	
Moderately active	320 (32.5)	535 (31.4)	
Vigorously active	291 (29.6)	295 (17.3)	
Extremely active	196 (19.9)	141 (8.3)	<0.001

Wilcoxon rank-sum for continuous and chi-square for categorical

Numbers may differ due to missing values; SD: standard deviation

Table S2. The five most common jobs, the five most heat exposed jobs and the five most common heat exposed jobs

	Job Code	Level (%)	Proportion (%)	Proportion of all jobs (%)
**Five most common jobs**				
Administrative assistants without front-office duties not classified above	4300	0	0	6.87
Shop assistants and display clerks in shops, stores, kiosks and markets	5330	0	0	6.31
Sales representatives and sales technicians	3320	0	0	3.55
Domestic workers	9110	0	0	2.91
Waiters, waitresses, bartenders and the like	5020	25	25	2.56
**Five most heat exposed jobs**				
Operators in ore furnaces and primary metal melting furnaces	8121	100	100	0.27
Operators in secondary melting furnaces, metal casting and moulding machines; rolling mill operators	8122	100	100	0.23
Operators of glassmaking and ceramics kilns and similar machinery	8131	100	100	0.14
Blacksmiths and smiths	7521	100	100	0.13
Boiler and steam engine operators	8162	100	100	0.10
**Five most common heat exposed jobs**				
Waiters, waitresses, bartenders and the like	5020	25	25	2.56
Skilled own-account workers in agricultural activities, except in orchards, nurseries and gardens	6011	35	100	2.22
Cooks and other food preparers	5010	60	70	1.78
Bricklayers and masons	7110	25	100	1.68
Labourers in manufacturing industries	9700	25	30	1.40

Table S3: Associations between colorectal cancer and occupational heat exposure for participants never/ever exposed to metals (OR: Odds Ratio; 95% CI: 95% Confidence Interval)

	**Never metals**		**Ever metals**		**P-values for interaction**
	**Cases/Controls (N)**	**OR (95% CI)**	**Cases/Controls (N)**	**OR (95% CI)**	
**Never heat exposure**	521/1,591	1 (ref)	64/113	1 (ref)	
**Ever heat exposure**	276/465	1.21 (0.98-1.49)	567/861	0.96 (0.68-1.36)	0.22
**Lifetime Cumulative Exposure**[a]					
Low	104/217	1.24 (0.94-1.63)	159/260	1.07 (0.72-1.57)	
Medium	92/155	1.12 (0.82-1.53)	195/320	0.92 (0.63-1.35)	
High	80/93	1.27 (0.88-1.82)	213/281	0.88 (0.60-1.31)	
P-trend		0.13		0.29	0.13
**Duration (Years)**[b]					
>0 - <15	121/256	1.23 (0.96-1.60)	156/276	0.96 (0.65-1.41)	
>= 15 - <30	50/91	1.07 (0.73-1.58)	121/172	1.05 (0.70-1.58)	
>=30	105/118	1.25 (0.90-1.75)	290/413	0.91 (0.63-1.33)	
P-trend		0.14		0.63	0.16

Adjusted for age, sex, region, education, cigarette smoking, family history of colorectal cancer, BMI and occupational physical activity

[a]P*L*duration in years, cut points based on those of the overall population

[b]Based on the tertiles according to the distribution amongst exposed controls

P-value of 5 for heat and metal exposure

*lead, cadmium, chromium, nickel, iron

Table S4. Associations between colorectal cancer and occupational heat exposure for participants never/ever exposed to solvents (OR: Odds Ratio; 95% CI: 95% Confidence Interval)

	**Never solvents**		**Ever solvents**		**P-values for interaction**
	**Cases/Controls (N)**	**OR (95% CI)**	**Cases/Controls (N)**	**OR (95% CI)**	
**Never heat exposure**	533/1,595	1 (ref)	52/110	1 (ref)	
**Ever heat exposure**	691/1,062	1.10 (0.92-1.30)	154/262	1.22 (0.79-1.87)	0.93
**Lifetime Cumulative Exposure**[a]					
Low	203/391	1.17 (0.95-1.45)	60/87	1.64 (0.99-2.72)	
Medium	234/352	1.08 (0.86-1.35)	55/122	0.88 (0.53-1.46)	
High	254/319	0.99 (0.77-1.26)	39/53	1.29 (0.71-2.33)	
P-trend		0.94		1.00	0.09
**Duration (Years)**[b]					
>0 - <15	214/424	1.15 (0.93-1.42)	63/109	1.31 (0.80-2.16)	
>= 15 - <30	130/193	1.15 (0.87-1.51)	42/69	1.30 (0.75-2.28)	
>=30	347/445	1.00 (0.80-1.25)	49/84	1.02 (0.59-1.76)	
P-trend		0.92		0.94	0.35

Adjusted for age, sex, region, education, cigarette smoking, family history of colorectal cancer, BMI and occupational physical activity

[a]P*L*duration in years, cut points based on those of the overall population

[b]Based on the tertiles according to the distribution amongst exposed controls

P-value of 5 for heat and solvent exposure

*aliphatic and alicyclic hydrocarbons, aromatic hydrocarbons, chlorinated hydrocarbons, and other organic solvents

Table S5. Associations between colorectal cancer and occupational heat exposure for participants never/ever exposed to pesticides (OR: Odds Ratio; 95% CI: 95% Confidence Interval)

	**Never Pesticides**		**Ever Pesticides**		**P-values for interaction**
	**Cases/Controls (N)**	**OR (95% CI)**	**Cases/Controls (N)**	**OR (95% CI)**	
**Never heat exposure**	543/1,617	1 (ref)	41/86	1 (ref)	
**Ever heat exposure**	533/862	1.12 (0.94-1.33)	307/464	1.27 (0.78-2.09)	0.46
**Lifetime Cumulative Exposure**[a]					
Low	198/340	1.28 (1.03-1.59)	64/138	1.19 (0.69-2.06)	
Medium	184/294	1.08 (0.85-1.37)	103/180	1.20 (0.69-2.08)	
High	151/228	0.89 (0.68-1.17)	140/146	1.60 (0.90-2.84)	
P-trend		0.74		0.09	0.09
**Duration (Years)**[b]					
>0 - <15	182/351	1.21 (0.97-1.50)	94/182	1.24 (0.74-2.06)	
>= 15 - <30	119/179	1.21 (0.91-1.60)	52/84	1.25 (0.67-2.32)	
>=30	232/332	0.96 (0.76-1.22)	161/198	1.45 (0.81-2.59)	
P-trend		0.97		0.24	0.40

Adjusted for age, sex, region, education, cigarette smoking, family history of colorectal cancer, BMI and occupational physical activity

[a]P*L*duration in years, cut points based on those of the overall population

[b]Based on the tertiles according to the distribution amongst exposed controls

P-value of 5 for heat and pesticide exposure

*2,4-D, atrazine, captan, chlorpyrifos, dicuat, diuron, endosulfan, methomyl, pyrethrin, tiram

Table S6. Associations between colorectal cancer and occupational heat exposure for participants never/ever exposed to polycyclic aromatic hydrocarbons (PAHs) (OR: Odds Ratio; 95% CI: 95% Confidence Interval)

	**Never PAHs**		**Ever PAHs**		**P-values for interaction**
	**Cases/Controls (N)**	**OR (95% CI)**	**Cases/Controls (N)**	**OR (95% CI)**	
**Never heat exposure**	551/1,646	1 (ref)	34/60	1 (ref)	
**Ever heat exposure**	637/1,009	1.14 (0.96-1.35)	204/317	1.00 (0.61-1.65)	0.58
**Lifetime Cumulative Exposure**[a]					
Low	205/402	1.18 (0.95-1.46)	56/76	1.40 (0.77-2.54)	
Medium	224/352	1.10 (0.88-1.38)	63/122	0.84 (0.48-1.48)	
High	208/255	1.11 (0.86-1.43)	85/119	0.91 (0.52-1.60)	
P-trend		0.35		0.34	0.10
**Duration (Years)**[b]					
>0 - <15	215/447	1.14 (0.93-1.40)	59/86	1.23 (0.68-2.21)	
>= 15 - <30	115/184	1.18 (0.89-1.56)	56/79	1.13 (0.63-2.04)	
>=30	307/378	1.11 (0.88-1.40)	89/152	0.81 (0.47-1.40)	
P-trend		0.30		0.21	0.18

Adjusted for age, sex, region, education, cigarette smoking, family history of colorectal cancer, BMI and occupational physical activity

[a]P*L*duration in years, cut points based on those of the overall population

[b]Based on the tertiles according to the distribution amongst exposed controls

P-value of 5 for heat and PAH exposure

Table S7. Associations between colorectal cancer and occupational heat exposure for participants never/ever exposed to detergents (OR: Odds Ratio; 95% CI: 95% Confidence Interval)

	**Never detergents**		**Ever detergents**		**P-values for interaction**
	**Cases/Controls (N)**	**OR (95% CI)**	**Cases/Controls (N)**	**OR (95% CI)**	
**Never heat exposure**	414/1,244	1 (ref)	171/456	1 (ref)	
**Ever heat exposure**	336/462	1.09 (0.87-1.38)	504/865	1.24 (0.97-1.58)	0.54
**Lifetime Cumulative Exposure[a]**					
Low	116/192	1.18 (0.89-1.57)	144/286	1.36 (1.02-1.82)	
Medium	117/153	1.09 (0.80-1.50)	170/322	1.10 (0.82-1.49)	
High	103/117	0.92 (0.65-1.32)	190/257	1.23 (0.90-1.68)	
P-trend		0.96		0.34	0.21
**Duration (Years)[b]**					
>0 - <15	91/163	1.15 (0.85-1.57)	182/370	1.27 (0.97-1.67)	
>= 15 - <30	59/90	1.08 (0.73-1.60)	113/173	1.36 (0.98-1.90)	
>=30	209/186	1.05 (0.78-1.40)	209/322	1.08 (0.79-1.48)	
P-trend		0.71		0.68	0.2

Adjusted for age, sex, region, education, cigarette smoking, family history of colorectal cancer, BMI and occupational physical activity

[a]P*L*duration in years, cut points based on those of the overall population

[b]Based on the tertiles according to the distribution amongst exposed controls

P-value of 5 for heat and detergent exposure

Table S8. Associations between occupational heat exposure and colorectal cancer stratified by time since last heat exposure (OR: Odds Ratio; 95% CI: 95% Confidence Interval)

	Last heat exposure ≥5 & <10 years ago		Last heat exposure ≥10 & <20 years ago		Last heat exposure ≥20 years ago	
	**Cases/Controls (N)**	**OR (95% CI)**	**Cases/Controls (N)**	**OR (95% CI)**	**Cases/Controls (N)**	**OR (95% CI)**
**Never heat exposure**	585/1,706	1 (ref)	585/1,706	1 (ref)	585/1,706	1 (ref)
**Ever heat exposure**	224/403	1.10 (0.87-1.39)	153/197	1.11 (0.84-1.47)	236/384	1.06 (0.85-1.32)
**Lifetime Cumulative Exposure**[a]						
Low	37/86	1.53 (0.99-2.34)	24/51	1.47 (0.87-2.47)	92/191	1.02 (0.77-1.35)
Medium	74/139	1.07 (0.76-1.51)	35/50	1.09 (0.68-1.76)	89/139	1.07 (0.78-1.46)
High	113/178	0.96 (0.71-1.30)	94/96	0.99 (0.69-1.42)	55/54	1.18 (0.76-1.81)
P-trend		0.97		0.80		0.47
**Duration (Years)**[b]						
>0 - <15	39/103	1.29 (0.85-1.94)	27/56	1.50 (0.90-2.48)	157/292	1.07 (0.84-1.35)
>= 15 - <30	50/85	1.34 (0.90-1.99)	29/37	1.28 (0.75-2.18)	40/63	0.86 (0.55-1.34)
>=30	135/215	0.95 (0.71-1.27)	97/104	0.92 (0.64-1.31)	39/29	1.41 (0.82-2.42)
P-trend		0.92		0.96		0.49

All models are adjusted for age, sex, region, education, cigarette smoking, family history of colorectal cancer, BMI and occupational physical activity

[a]P*L*duration in years, cut points based on those of the controls overall

[b]Based on the tertiles according to the distribution amongst exposed controls

Table S9. Association between occupational heat exposure and colorectal cancer using different combinations of lag years, durations and proportion thresholds

	**P-threshold 5 & duration at least 1 year & lag 1 year**	**P-threshold 5 & duration at least 5 years & lag 1 year**	**P-threshold 25 & duration at least 1 year & lag 1 year**	**P-threshold 25 & duration at least 5 years & lag 1 year**	**P-threshold 50 & duration at least 1 year & lag 1 year**	**P-threshold 50 & duration at least 5 years & lag 1 year**
	**OR (95% CI)**	**OR (95% CI)**	**OR (95% CI)**	**OR (95% CI)**	**OR (95% CI)**	**OR (95% CI)**
**Controls/cases (N)**	3,045/1,434	2,837/1,335	2,697/1,200	2,518/1,121	2,366/1,032	2,260/980
**Never heat exposure**	1 (ref)	1 (ref)	1 (ref)	1 (ref)	1 (ref)	1 (ref)
**Ever heat exposure**	1.12 (0.96-1.31)	1.11 (0.94-1.32)	1.09 (0.92-1.29)	1.09 (0.91-1.31)	1.07 (0.88-1.29)	1.06 (0.87-1.30)
**Lifetime Cumulative Exposure[a]**						
Low	1.21 (1.00-1.48)	1.24 (0.98-1.57)	1.15 (0.91-1.44)	1.21 (0.89-1.63)	1.11 (0.81-1.51)	1.09 (0.68-1.74)
Medium	1.06 (0.86-1.30)	1.07 (0.87-1.32)	1.07 (0.85-1.35)	1.08 (0.85-1.36)	1.06 (0.82-1.38)	1.08 (0.83-1.41)
High	1.05 (0.84-1.31)	1.05 (0.84-1.31)	1.04 (0.83-1.31)	1.04 (0.82-1.31)	1.05 (0.82-1.34)	1.04 (0.81-1.34)
P-trend	0.67	0.68	0.63	0.65	0.65	0.66
**Duration (Years)[b]**						
>0 - <15	1.18 (0.98-1.43)	1.20 (0.96-1.51)	1.14 (0.93-1.40)	1.19 (0.93-1.52)	1.06 (0.83-1.35)	1.05 (0.77-1.42)
>= 15 - <30	1.10 (0.86-1.41)	1.10 (0.86-1.41)	1.09 (0.83-1.45)	1.09 (0.83-1.45)	1.15 (0.83-1.59)	1.15 (0.83-1.59)
>=30	1.05 (0.86-1.29)	1.05 (0.85-1.29)	1.01 (0.80-1.27)	1.00 (0.80-1.27)	1.04 (0.81-1.33)	1.03 (0.80-1.33)
P-trend	0.66	0.67	0.84	0.88	0.64	0.69

All models are adjusted for age, sex, region, education, cigarette smoking, family history of colorectal cancer, BMI and occupational physical activity

[a]P*L*duration in years, cut points based on those of the controls overall

[b]Based on the tertiles according to the distribution amongst exposed controls

Table S10. Association between occupational heat exposure and colorectal cancer using different combinations of lag years, durations and proportion thresholds

	P-threshold 5 & duration at least 1 year & lag 5 years	P-threshold 5 & duration at least 5 years & lag 5 years	P-threshold 25 & duration at least 5 years & lag 5 years	P-threshold 50 & duration at least 1 year & lag 5 years	P-threshold 50 & duration at least 5 years & lag 5 years
	**OR (95% CI)**	**OR (95% CI)**	**OR (95% CI)**	**OR (95% CI)**	**OR (95% CI)**
**Controls/cases (N)**	3,033/1,430	2,819/1,328	2,501/1,117	2,365/1,033	2,250/980
**Never heat exposure**	1 (ref)	1 (ref)	1 (ref)	1 (ref)	1 (ref)
**Ever heat exposure**	1.12 (0.96-1.31)	1.12 (0.95-1.33)	1.10 (0.92-1.33)	1.06 (0.88-1.29)	1.08 (0.88-1.32)
**Lifetime Cumulative Exposure[a]**					
Low	1.24 (1.03-1.51)	1.29 (1.02-1.63)	1.26 (0.93-1.70)	1.11 (0.82-1.51)	1.17 (0.73-1.87)
Medium	1.03 (0.84-1.26)	1.05 (0.85-1.30)	1.08 (0.86-1.37)	1.08 (0.83-1.40)	1.11 (0.85-1.45)
High	1.03 (0.83-1.29)	1.04 (0.83-1.30)	1.04 (0.82-1.31)	1.02 (0.79-1.31)	1.02 (0.79-1.32)
P-trend	0.83	0.77	0.64	0.74	0.70
**Duration (Years)[b]**					
>0 - <15	1.18 (0.97-1.42)	1.20 (0.95-1.50)	1.20 (0.94-1.53)	1.05 (0.82-1.33)	1.07 (0.79-1.44)
>= 15 - <30	1.16 (0.92-1.48)	1.17 (0.92-1.50)	1.12 (0.85-1.48)	1.18 (0.86-1.63)	1.18 (0.85-1.63)
>=30	1.02 (0.83-1.25)	1.02 (0.83-1.26)	1.01 (0.80-1.28)	1.03 (0.79-1.33)	1.03 (0.79-1.33)
P-trend	0.78	0.73	0.81	0.66	0.67

All models are adjusted for age, sex, region, education, cigarette smoking, family history of colorectal cancer, BMI and occupational physical activity

[a]P*L*duration in years, cut points based on those of the controls overall

[b]Based on the tertiles according to the distribution amongst exposed controls

Table S11. Association between occupational heat exposure and colorectal cancer using different combinations of lag years, durations and proportion thresholds

	**P-threshold 5 & duration at least 1 year & lag 10 years**	**P-threshold 5 & duration at least 5 years & lag 10 years**	**P-threshold 25 & duration at least 1 year & lag 10 years**	**P-threshold 25 & duration at least 5 years & lag 10 years**	**P-threshold 50 & duration at least 1 year & lag 10 years**	**P-threshold 50 & duration at least 5 years & lag 10 years**
	**OR (95% CI)**	**OR (95% CI)**	**OR (95% CI)**	**OR (95% CI)**	**OR (95% CI)**	**OR (95% CI)**
**Controls/cases (N)**	2,990/1,418	2,776/1,311	2,653/1,193	2,467/1,105	2,342/1,033	2,228/976
**Never heat exposure**	1 (ref)	1 (ref)	1 (ref)	1 (ref)	1 (ref)	1 (ref)
**Ever heat exposure**	1.13 (0.96-1.32)	1.10 (0.93-1.31)	1.11 (0.93-1.31)	1.09 (0.90-1.31)	1.10 (0.91-1.33)	1.08 (0.88-1.33)
**Lifetime Cumulative Exposure**[a]						
Low	1.22 (1.01-1.48)	1.23 (0.97-1.55)	1.14 (0.90-1.43)	1.12 (0.82-1.52)	1.11 (0.82-1.51)	1.08 (0.67-1.72)
Medium	1.07 (0.87-1.31)	1.07 (0.86-1.32)	1.15 (0.91-1.44)	1.15 (0.91-1.45)	1.19 (0.92-1.54)	1.19 (0.91-1.55)
High	1.03 (0.82-1.30)	1.02 (0.81-1.29)	1.02 (0.81-1.30)	1.01 (0.79-1.29)	1.02 (0.79-1.31)	1.00 (0.77-1.29)
P-trend	0.74	0.83	0.56	0.67	0.57	0.70
**Duration (Years)**[b]						
>0 - <15	1.18 (0.98-1.43)	1.16 (0.93-1.46)	1.14 (0.93-1.40)	1.13 (0.88-1.44)	1.12 (0.88-1.43)	1.12 (0.83-1.50)
>= 15 - <30	1.21 (0.96-1.53)	1.22 (0.96-1.54)	1.23 (0.94-1.61)	1.23 (0.94-1.61)	1.18 (0.87-1.61)	1.18 (0.86-1.61)
>=30	0.98 (0.79-1.22)	0.97 (0.78-1.21)	0.96 (0.76-1.23)	0.95 (0.74-1.21)	1.02 (0.78-1.33)	1.00 (0.76-1.31)
P-trend	0.91	0.98	0.85	0.98	0.63	0.77

All models are adjusted for age, sex, region, education, cigarette smoking, family history of colorectal cancer, BMI and occupational physical activity

[a]P*L*duration in years, cut points based on those of the controls overall

[b]Based on the tertiles according to the distribution amongst exposed controls
